# Characterization of the Mechanical Behavior and Stabilization Mechanism of Soft Soil Treated with Xanthan Gum Biopolymer

**DOI:** 10.3390/polym17111532

**Published:** 2025-05-30

**Authors:** Qian-Feng Gao, Xue-Ke Shi, Ling Zeng, Hui-Cong Yu, Jun-Xia Hu

**Affiliations:** 1 School of Transportation, Changsha University of Science & Technology, Changsha 410114, China; shixueke@stu.csust.edu.cn; 2 School of Civil and Environmental Engineering, Changsha University of Science & Technology, Changsha 410114, China; zl001@csust.edu.cn (L.Z.); yuhuicong@stu.csust.edu.cn (H.-C.Y.); 3 Laboratoire de Génie Civil et géo-Environnement, Université de Lille, 59370 Lille, France; 4 College of Ship and Architecture, Jiaxing Nanyang Polytechnic Institute, Jiaxing 314031, China; hujunxia2025@163.com

**Keywords:** soft soil, soil stabilization, xanthan gum, unconfined compressive strength, stabilization mechanism

## Abstract

Soft soil poses significant challenges in highway engineering due to its low strength and high compressibility. This study proposes using xanthan gum biopolymer as an environmentally friendly agent to improve the mechanical behavior of soft soil. Laboratory tests were conducted to analyze the unconfined compressive strength (UCS) and compressibility of xanthan-gum-stabilized soft soil under dry–wet cycles. Physicochemical analysis was performed to examine the pH value, electrical conductivity, and total dissolved solids (TDS) of the stabilized soil. Additionally, microscopic tests were performed to investigate the stabilization mechanism. The results demonstrate that the UCS of the stabilized soil consistently increases with curing age while it decreases under dry–wet cycles. Moreover, the UCS, durability, and modulus of compressibility of the stabilized soil initially increase significantly and then slightly decrease with increasing xanthan gum dosage. At the optimal xanthan gum dosage (1.5%), the UCS reaches 376.3 kPa at 28 d of curing and drops by only 24.1% even after ten dry–wet cycles, and the modulus of compressibility is enhanced to 37.13 MPa; meanwhile, the corresponding compression index and coefficient of compressibility are reduced to 0.082 and 0.061 MPa^−1^, respectively, indicating satisfactory performance of the stabilized soil as highway foundation material. The stabilization mechanism of xanthan-gum-treated soft soil primarily involves the bonding and filling effects of the hydrogel resulting from the hydration of xanthan gum. These findings suggest that xanthan gum is a promising and effective stabilizing agent for soft soil as it can significantly reduce soil water content and void ratio.

## 1. Introduction

Highways, integral components of the transportation network, serve as vital lifelines for economic development, playing a significant role in propelling national economy growth. However, in many coastal and riverine regions, a wide array of soft soil layers is commonly encountered. These soft soil deposits exhibit undesirable engineering characteristics, including high water contents, large void ratios, substantial compressibility, and low bearing capacity [1,2]. As a result, constructing highways in such soft soil regions without stabilization can lead to numerous engineering issues, such as foundation failures, excessive embankment settlements, and slope instability [3,4,5]. Replacing the soft soil layer with superior materials would considerably increase project costs, thereby reducing economic benefits. Moreover, the disposal of excavated soft soil would occupy valuable land space and contribute to environmental pollution. This raises the need for stabilizing soft soil to improve its engineering properties and ensure its capability for highway foundations.

Soil stabilization is one of the most widely used methods for soft soil reinforcement. Its fundamental principle involves integrating stabilizing agents into soft soil to transform it into a solidified matrix, thereby substantially enhancing the soil strength and stability. Soil stabilization can be categorized into mechanical, chemical, and biological methods based on the agent types. Mechanical stabilization improves soil behavior by modifying its physical properties and typically includes techniques such as soil compaction, grading improvement, and geotextile reinforcement [4,6,7]. This method often yields immediate soil improvement and is cost-effective and applicable to various soil types and conditions. However, it also exhibits limitations such as reduced effectiveness at greater depths, potential environmental disturbance from heavy machinery, as well as the necessity for ongoing maintenance to sustain improvements over time. Chemical stabilization lies in modifying soil properties through physicochemical reactions between soil and chemical agents. Lime and cement are two traditional and widely utilized chemical agents for soil stabilization. Many previous studies have confirmed their efficacy in enhancing the compressive strength, shear strength, bearing capacity, and resistance to compressibility of soft soil [8,9,10]. However, a significant drawback of these agents lies in the substantial carbon dioxide emissions generated during their production. Therefore, it is crucial to minimize the use of lime and cement to alleviate their environmental impact. For this purpose, various additives such as industrial wastes (e.g., fly ash and steel slag), fibers (e.g., natural or synthetic fibers), and nanomaterials (e.g., SiO_2_, TiO_2_, and Al_2_O_3_) are incorporated to reduce lime/cement usages while promoting their hydration process [11,12,13,14,15]. Nevertheless, industrial wastes may contain pollutants (e.g., heavy metals and toxins), posing potential environmental and ecological risks. Moreover, the agglomeration problem with fibers and nanomaterials limits their effectiveness and applicability.

In recent years, biostabilization using biopolymers (e.g., xanthan gum, sodium alginate, and lignin) has gained increasing attention due to its environmental benefits [16,17,18,19]. Xanthan gum is one of the most commonly utilized biopolymers because it possesses high viscosity, distinctive rheological properties, excellent water solubility, as well as remarkable thermal and pH stability [20,21]. Several researchers have investigated the effect of xanthan gum on the mechanical behavior of various soils, including kaolin, expansive soil, red clay, silt, and sand [22,23]. It is reported that adding xanthan gum can significantly increase the unconfined compressive strength (UCS) and shear strength of soil, and the optimal xanthan gum dosage is 1.0–2.0% depending on soil types [19,24]. Wang et al. [24] explained that xanthan gum enhances soil strengths by effectively wrapping soil particles and filling pore spaces; nevertheless, excessive xanthan gum may weaken the cohesive bond between soil particles. The stabilization effect of xanthan gum is affected by soil water content. Bozyigit et al. [25] showed that the maximum UCS of stabilized kaolin occurred at the optimum water content, and an increasing water content caused more ductile soil behavior. Chen et al. [26] and Zhang et al. [20] reported that the improvement effect of xanthan gum was obvious at a lower water content but decreases gradually with increasing water content. Xanthan gum also effectively enhances soil brittleness and reduces compressibility of various soils [19,27]. However, Vydehi and Moghal [28] noted that xanthan gum could potentially enhance the compressibility and swelling characteristics of expansive soil. Zhang et al. [20] evaluated the shear strength of xanthan-gum-treated silt under drying and wetting conditions. Their findings offer valuable guidance for the design and construction of geotechnical structures in silt-rich regions.

Despite its proven effectiveness in treating various soil types, the applicability of xanthan gum for stabilizing soft soil with extremely high water content remains uncertain. Specifically, there is a research gap regarding the impact of xanthan gum dosage on the compressibility and durability of soft soil. Considering the inherent characteristics of both soft soil and xanthan gum, we hypothesize that xanthan gum is particularly effective for stabilizing soft soil, as it can potentially reduce the soil water content and void ratio, which are two key factors contributing to the poor mechanical performance of soft soil.

The primary objective of this study is to evaluate the efficacy of using xanthan gum to improve the mechanical behavior of soft soil. To this end, geotechnical tests were conducted to analyze the UCS and compressibility of xanthan-gum-stabilized soft soil under dry–wet cycles. Subsequently, physicochemical analysis was conducted to examine the pH value, electrical conductivity, and total dissolved solids (TDS) of the stabilized soil. Finally, microscopic techniques such as Fourier transform infrared spectroscopy (FTIR), scanning electron microscopy (SEM), and energy-dispersive X-ray spectroscopy (EDS) were utilized to investigate the microscopic mechanism underlying the stabilization of soft soil by xanthan gum. The findings would offer critical guidance on stabilizing soft soil encountered during highway constructions, especially through the addition of environmentally sustainable agents.

## 2. Materials and Methods

### 2.1. Materials

#### 2.1.1. Soft Soil

**Figure 1:**
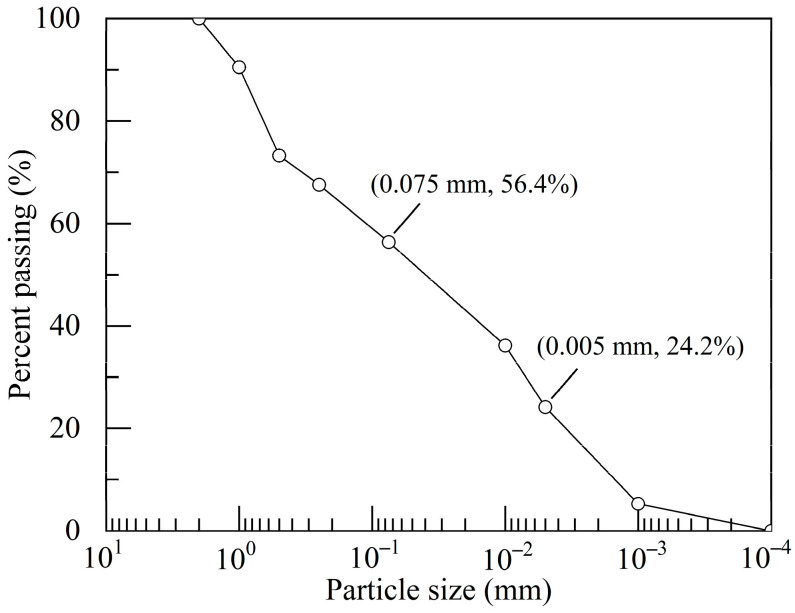
Particle size distribution of soft soil.

**Figure 2:**
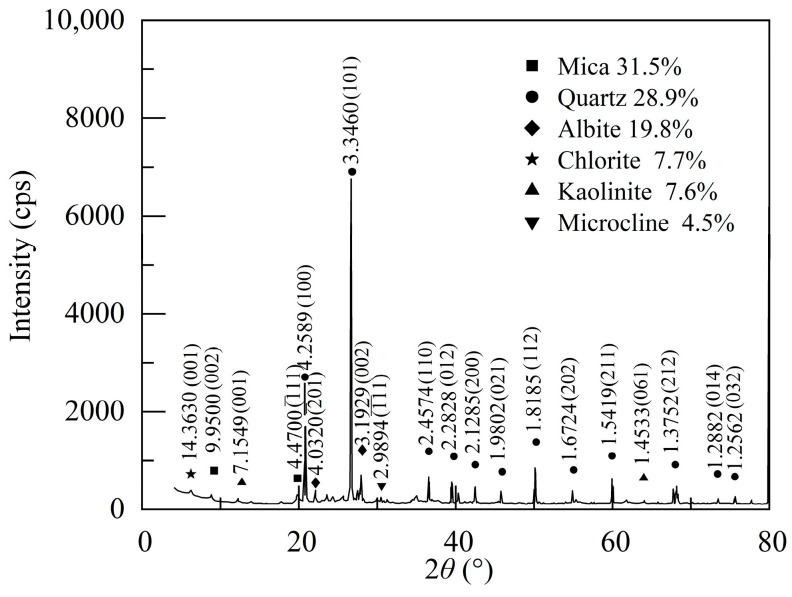
X-ray diffraction pattern of soft soil.

The soft soil used in this study was sourced from a lacustrine region in Nanyang City, Henan Province, China. This soil appears in a flow plastic state and displays a grayish-yellow hue. Its natural water content was 50.1% and natural density was 1.73 g/cm^3^, with a pH value of approximately 7.17. After drying and grinding, the soil samples successively underwent sieve analysis and laser particle size analysis. The results showed the mass fractions of clay particles, silt particles, and sand particles at 24.2%, 32.2%, and 43.6%, respectively (Figure 1). X-ray diffraction analysis revealed that the primary mineral components of the soil comprised mica (31.5%), quartz (28.9%), albite (19.8%), chlorite (7.7%), kaolinite (7.6%), and microcline (4.5%) (Figure 2). Laboratory geotechnical tests were conducted on soil particles smaller than 2.0 mm, yielding the key physical properties of the soft soil, as shown in Table 1. The soil’s specific gravity was 2.75, with a plastic limit of 22.9% and a liquid limit of 38.5%. The organic matter content determined by the dichromatometric method was 1.08%.

#### 2.1.2. Xanthan Gum

Xanthan gum is a polysaccharide biopolymer produced through fermentation by *Xanthomonas campestris* using glucose and sucrose as a carbon source. When properly mixed with water, it produces a stable, viscous water gel even at low concentrations. Xanthan gum was chosen as the stabilizing agent because of its proven high efficiency in soil stabilization, environmentally friendly characteristics, and cost-effectiveness. The xanthan gum used in this study was produced by Inner Mongolia Fufeng Biotechnologies Co., Ltd. (Hohhot, China). The fundamental properties of xanthan gum are presented in Table 2. It has a viscosity of approximately 1593 mPa·s and an absolute molecular mass of 6.64 × 10^−18^ g.

### 2.2. Experimental Methods

#### 2.2.1. Mechanical Tests

Unconfined compression tests and consolidation tests were conducted to comprehensively analyze the UCS, durability, and compressibility of xanthan-gum-stabilized soft soil.

Dry soil powder was taken and mixed with distilled water to prepare a slurry with a water content equal to the natural water content (i.e., 50.1%) of the soil. The slurry was then sealed in a plastic bag and allowed to stand for 24 h to ensure a uniform distribution of moisture in the soil. Due to the potential challenges in soil mixing caused by the high viscosity of xanthan gum gel when excessively used, the maximum xanthan gum dosage was set at 2.0%. Hence, eight different xanthan gum dosages (i.e., 0.25%, 0.50%, 0.75%, 1.00%, 1.25%, 1.50%, 1.75%, and 2.00%) were employed in specimen preparations. The xanthan gum dosage was defined as the mass ratio of the xanthan gum powder to the soft soil slurry. The xanthan gum powder was manually mixed with the soft soil slurry at different dosages. Subsequently, the mixture was stirred at a rate of 100 r/min for 30 min using an electric stirrer to ensure thorough uniformity. Following thorough mixing, the mixture was poured into cylindrical molds (with an inner diameter of 50 mm and a height of 100 mm). After compaction, the specimens were wrapped with plastic film and placed under standard conditions (i.e., temperature = 20 °C ± 2 °C and relative humidity ≥ 95%) for curing. Different curing ages of 3, 7, 14, 21, and 28 d were considered in the experiment. Note that the molds were kept in place during curing and removed only after the curing process to minimize the potential disturbance of the specimens. Three parallel specimens were prepared and tested for each xanthan gum dosage and curing age condition.

**Figure 3:**
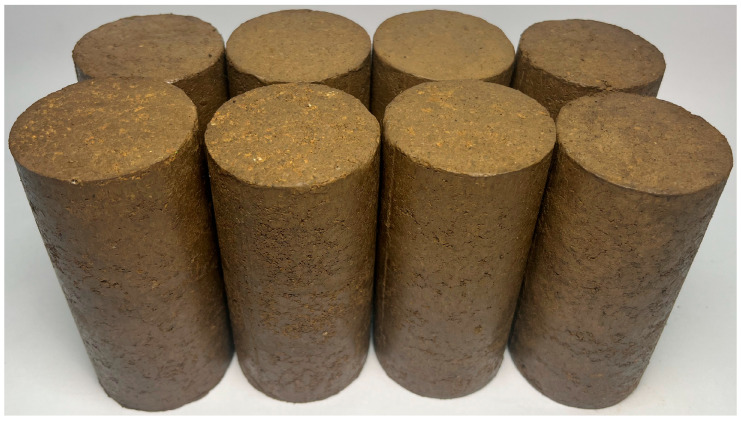
Xanthan-gum-stabilized soil specimens after demolding.

**Figure 4:**
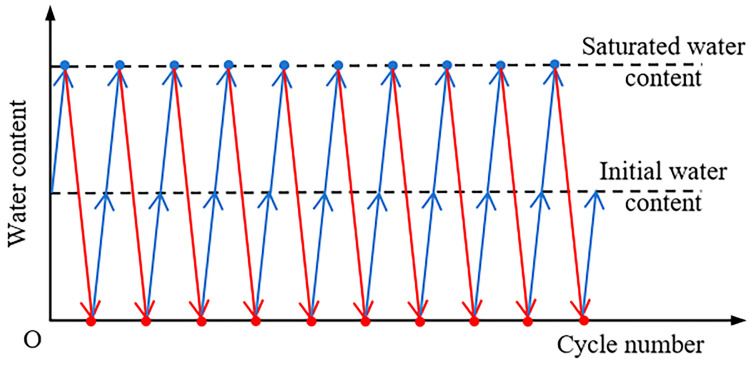
Schematic of the applied dry–wet cycles.

After curing, unconfined compression tests were conducted on the stabilized soil specimens with a diameter of 50 mm and a height of 100 mm (Figure 3) using a universal testing machine at a loading rate of 1.0% per minute. The procedures for unconfined compression tests referred to the Chinese specification (JTG 3430-2020) [29]. Additionally, the UCS the stabilized soil specimens with a curing age of 28 d subjected to dry–wet cycles was tested to examine the durability of xanthan-gum-stabilized soft soil. Multiple numbers of dry–wet cycles (i.e., 0, 2, 4, 6, 8, and 10 cycles) were applied in the tests. During the drying process, the specimens were dried at a low temperature of 40 °C in an oven, and their mass was measured every two hours. If the mass difference between two consecutive measurements was less than 0.1 g, the specimens were considered to have reached a dry state. The wetting process was carried out using the water film transfer method [30,31]. After determining the amount of water required for the specimens to reach saturation, the water was slowly dripped into a glass beaker containing the specimens. When the specimens had fully absorbed the water, they were considered to have reached the saturated state. Figure 4 illustrates the evolution in water content of the specimens during dry–wet cycles.

One-dimensional consolidation tests were conducted on xanthan-gum-stabilized soil specimens (61.8 mm in diameter and 20 mm in height) in strict accordance with the Chinese specification (JTG 3430-2020). Eight loading levels at 12.5, 25, 50, 100, 200, 300, 400, and 600 kPa were applied as the vertical pressure. Each loading level was sustained for 24 h or until the deformation increment of the specimen reached a value below 0.01 mm/h. Finally, the compression parameters, including the compression index, modulus of compressibility (*E_s_*), and coefficient of compressibility (*a_s_*), were determined based on the test results. The consolidation yield stress of the specimens was obtained according to the Casagrande graphical method [32].

#### 2.2.2. Physicochemical Tests

The purpose of this section is to analyze the influence of xanthan gum dosages on the physicochemical properties of soft soil. Electrical conductivity, pH, and TDS tests were conducted on xanthan-gum-stabilized soil specimens cured for 7 d following different technical codes (JTG 3430-2020; HJ802-2016) [29,33]. Initially, the cylindrical specimens with various xanthan gum dosages after unconfined compression tests were dried in a 40 °C oven for 48 h. Subsequently, they were ground to obtain dry soil powder with particle sizes less than 0.075 mm. A 10 g portion of the powder was added to a beaker containing 50 mL distilled water, and the mixture was then stirred centrifugally to form soil suspensions. Finally, the pH value was measured using a PHS-3E precision pH meter, and the electrical conductivity and TDS were measured using a STARTER3100C conductivity meter (OHAUS, Parsippany, NJ, USA).

#### 2.2.3. Microscopic Tests

FTIR tests and SEM-EDS tests were performed to examine the functional groups, microstructure, and chemical compositions of soft soil before and after stabilization with xanthan gum. The specimens cured to the designated age were oven-dried at 65 °C to a constant weight. A small cube (20 mm × 20 mm × 20 mm) was then extracted from the central region of each specimen. After careful grinding and sieving, soil powder with particle sizes less than 0.075 mm was used for FTIR testing. The instrument employed was the Nicolet iS50 FTIR Spectrometer, manufactured by Thermo Fisher Scientific Inc. (Waltham, MA, USA). The powder samples were scanned from 4000 to 400 cm^−1^ wavenumbers at a scan rate of 2 mm/s with a resolution of 4 cm^−1^. The samples for SEM-EDS tests had dimensions of 10 mm × 10 mm × 5 mm and were extracted from the central portion of the cylindrical specimens after unconfined compression tests [34]. These extracted samples were initially dehydrated in a low-temperature (40 °C) oven for 24 h. Afterward, the horizontal surfaces of the samples were chosen as the observation planes and coated with thin layers of gold under vacuum using the 108 auto sputter coater (Cressington Scientific Instruments, Watford, UK). Finally, the samples were transferred into the SEM chamber for both SEM observation and EDS analysis. The testing instrument employed was the ZEISS EVO MA10 microscope, manufactured by ZEISS Group (Oberkochen, Germany).

## 3. Results

### 3.1. Unconfined Compressive Behavior

#### 3.1.1. Stress–Strain Relationship

**Figure 5:**
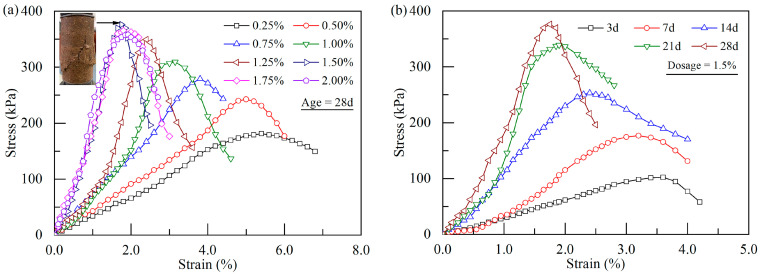
Stress–strain curves of xanthan-gum-stabilized soft soil: (**a**) effect of xanthan gum dosage; (**b**) effect of curing age.

Figure 5 shows the stress–strain curves of stabilized soft soil specimens at different xanthan gum dosages and curing ages. It is noted that, with an increase in axial strain, the stress of the specimen presents a significant increase during the initial phase, followed by a notable drop upon reaching the peak, exhibiting a typical strain-softening behavior. At a dosage below 1.0%, the specimen shows a small peak stress with considerable deformation, spanning an axial strain range from 0.0% to 7.0%. However, as the xanthan gum dosage exceeds 1.0%, the stress–strain curve gradually exhibits a distinct peak with less deformation (the corresponding axial strain ranges from 1.75% to 1.92%). This phenomenon becomes particularly pronounced at a xanthan gum dosage of 1.5%. Additionally, the curing age plays a significant role in shaping the stress–strain curve of the stabilized soil (Figure 5b). In general, on older curing age results in a higher peak stress, accompanied by a smaller strain at the peak stress.

#### 3.1.2. Unconfined Compressive Strength

**Figure 6:**
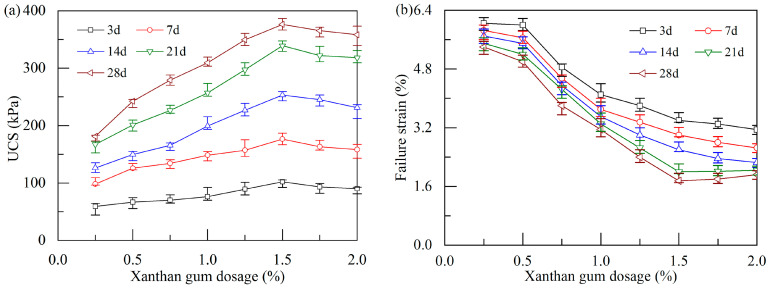
Effect of xanthan gum dosage and curing age on the UCS and failure strain: (**a**) UCS; (**b**) failure strain.

Figure 6a illustrates the combined effects of xanthan gum dosage and curing age on the UCS of the stabilized soil. It shows that the growth in the UCS of soft soil after stabilization depends on both the xanthan gum dosage and curing age.

At a fixed xanthan gum dosage, the UCS of the stabilized soil shows a positive correlation with the curing age. For instance, the UCS of the stabilized soil with a dosage of 1.5% cured for 28 d shows an increase of up to 2.69 times compared with that cured for 3 d, suggesting that prolonging the curing age contributes to increasing the strength of the stabilized soil. The above finding is consistent with the results of Bozyigit et al. [25], who reported that the strength of xanthan-gum-stabilized kaolin increases with curing age, and a 3-year cured specimen could show strength three times greater than that of a specimen cured for 90 d. This long-term growth behavior in soil strength demonstrates a significant advantage of using xanthan gum for soil stabilization.

Moreover, at the same curing age, the UCS of the stabilized soil exhibits an initial increase followed by a slight decrease with increasing xanthan gum dosage. Notably, a significant rise in the UCS is observed as the xanthan gum dosage increases from 0.0% to 1.5%. However, once the xanthan gum dosage exceeds 1.5%, a slight reduction in the UCS is observed. The phenomenon may be attributed to the fact that xanthan gum molecular chains tend to entangle at higher dosages, leading to localized thickening and uneven distribution of the hydrogel [35]. This finding underscores that the optimal xanthan gum dosage is 1.5% (corresponding to 25.95 kg xanthan gum for treating 1 m^3^ soft soil), at which the stabilized soil possesses the highest UCS (e.g., 376.3 kPa at 28 d), indicating the remarkable efficacy of xanthan gum in improving the strength of soft soil. A UCS value of 376.3 kPa not only satisfies the basic requirements for highway foundations but also meets the requirements for embankment fillers, as specified in the Chinese technical code T/CHCA 001-2023 [36].

The axial strain of the stabilized soil corresponds to the peak stress in the stress–strain curve defined as the failure strain. The variation in failure strain at different xanthan gum dosages and curing ages is plotted in Figure 6b. When the curing age is less than or equal to 14 d, the failure strain progressively decreases with increasing xanthan gum dosage. However, when the curing age exceeds 14 d, the failure strain notably decreases and then slightly increases with an increase in xanthan gum dosage. At a curing age of 28 d, the stabilized soil with 1.5% xanthan gum dosage exhibits the minimum failure strain. This phenomenon suggests that the application of xanthan gum in soft soil tends to reduce its ductility while simultaneously enhancing its strength, as evidenced by the failure mode of the specimen (Figure 5a).

Compared with conventional chemical stabilizers such as cement, xanthan gum generally yields lower UCS but significantly enhances the ductility of the treated soil. Moreover, as a biopolymer, xanthan gum offers notable environmental advantages over cement, which is associated with high CO_2_ emissions [23,37].

#### 3.1.3. Durability Under Dry–Wet Cycles

**Figure 7:**
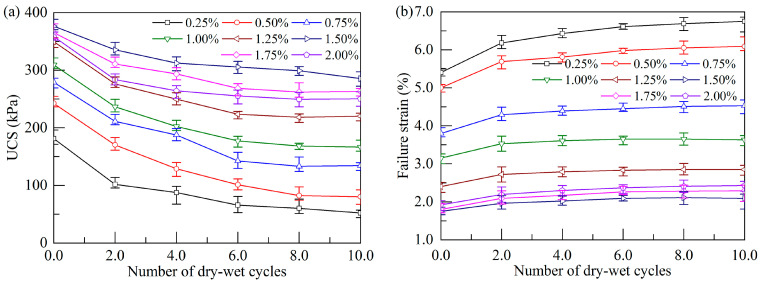
Effect of dry–wet cycles on UCS and failure strain at curing age of 28 d: (**a**) UCS; (**b**) failure strain.

The influence of dry–wet cycles on the UCS and failure strain of the stabilized soil at a curing age of 28 d is illustrated in Figure 7. As the cycle number increases, the UCS of the stabilized soil with different xanthan gum dosages generally experiences an initial sharp decline during the initial four cycles, followed by a stabilization trend thereafter (Figure 7a). Similar results were reported by Chen et al. [38], who found that the UCS of xanthan-gum-treated soil tended to stabilize after four cycles. They concluded that earlier dry–wet cycles have more pronounced negative impacts on the mechanical properties of the soil than later cycles. However, despite most of the strength reduction occurring during the initial cycles, the long-term durability performance beyond ten cycles remains an important and worthwhile direction for future investigation.

When the xanthan gum dosages are 0.25%, 0.75%, and 1.5%, the UCS of the specimens after ten dry–wet cycles decreases by 71.2%, 51.9%, and 24.1%, respectively. This result indicates that the rate of UCS degradation of the stabilized soil due to dry–wet cycles decreases gradually as the xanthan gum dosage increases. Nevertheless, when the xanthan gum dosage rises to 1.75% and 2.0%, the UCS after ten dry–wet cycles decreases by 27.9% and 30.2%, respectively. In other words, beyond a xanthan gum dosage of 1.5%, the rate of UCS gradation caused by dry–wet cycles slightly increases. Therefore, a xanthan gum dosage of 1.5% yields the optimal durability for xanthan-gum-stabilized soft soil under cyclic dry–wet conditions. The above findings are generally consistent with the results reported by Fatehi et al. [39] on kaolin–sand mixtures.

**Figure 8:**
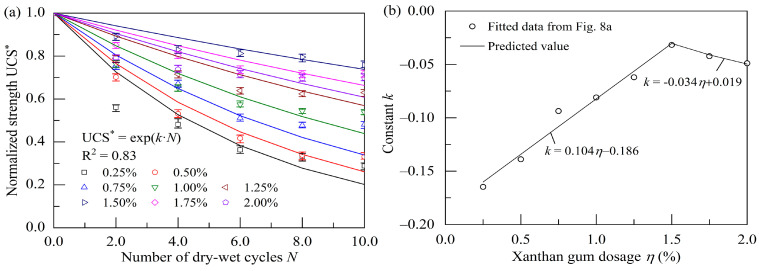
Effect of dry–wet cycles on normalized UCS at curing age of 28 d: (**a**) normalized UCS; (**b**) equation constant.

The UCS values of the stabilized soil after undergoing different dry–wet cycles are normalized by dividing them by the UCS of the specimens without experiencing dry–wet cycles. The change in the normalized UCS (UCS*) of the stabilized soil during dry–wet cycles is shown in Figure 8a. One can note that the normalized UCS can be expressed as an exponential function of the cycle number (*N*). The equation constants (*k*) corresponding to various xanthan gum dosages are plotted in Figure 8b. It shows that the relationship between the equation constant (*k*) and xanthan gum dosage (*η*) can be described by a piecewise linear function with the peak at a xanthan gum dosage of 1.5%:

(1) $${\mathrm{UCS}}^{*}=\exp(k\cdot N)$$

(2) $${\mathrm{UCS}}^{*}=\mathrm{UCS}/{\mathrm{UCS}}_{0}$$

(3) k=a⋅η+b

where UCS* is the normalized UCS; UCS_0_ is the UCS at zero dry–wet cycles; *N* is the number of dry–wet cycles; *η* is the xanthan gum dosage; *k*, *a*, and *b* are equation constants.

Dry–wet cycles also significantly affect the ductility of the stabilized soil. As shown in Figure 7b, with an increase in the number of dry–wet cycles, the failure strain of the stabilized soil monotonically increases, indicating an increase in soil ductility. However, after four dry–wet cycles, the increase in failure strain becomes minimal, presenting a stabilization trend in soil ductility. These results suggest that a moderate addition of xanthan gum significantly delays the strength degradation and ductility rise of the stabilized soil, thereby showing desirable durability under dry–wet cycles.

### 3.2. Consolidation Behavior

#### 3.2.1. Consolidation Curve

**Figure 9:**
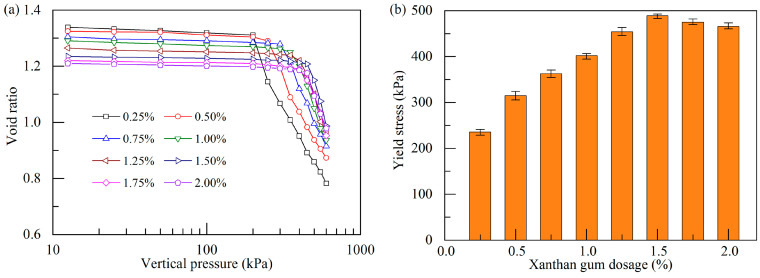
Effect of xanthan gum dosages on consolidation curve and yield stress: (**a**) consolidation curves; (**b**) consolidation yield stress.

Figure 9a illustrates the void ratio–vertical pressure curves of the stabilized soil with different xanthan gum dosages after curing for 28 d. It is noted that the initial void ratio of the stabilized soil gradually decreases with increasing xanthan gum dosage, suggesting that the addition of xanthan gum effectively reduces the void ratio of the stabilized soil. The consolidation curves in Figure 9a exhibit distinct two-stage features. As the vertical pressure increases, the void ratio first slightly decreases, showing minimal compressive deformation of the stabilized soil at a small vertical pressure. However, as the vertical pressure further increases, the void ratio drops greatly, exhibiting a significant compressive deformation at a high vertical pressure. This phenomenon occurs because the vertical load exceeds the consolidation yield stress formed by xanthan gum stabilization, leading to internal structural damage of the soil. Thus, there is a distinct yielding point in the consolidation curve, and this point is highly dependent on the xanthan gum dosage.

Figure 9b shows the influence of xanthan gum dosages on the consolidation yield stresses of the stabilized soil specimens. It is observed that the consolidation yield stress of the stabilized soil exhibits an initial increase followed by a slight decrease with increasing xanthan gum dosage. This phenomenon is similar to the evolution of UCS at different xanthan gum dosages. A comparison between the consolidation yield stress and UCS (Figure 6a) of the stabilized soil reveals that the ratio of the consolidation yield stress to the UCS falls between 1.2 and 1.3.

#### 3.2.2. Soil Compressibility

**Figure 10:**
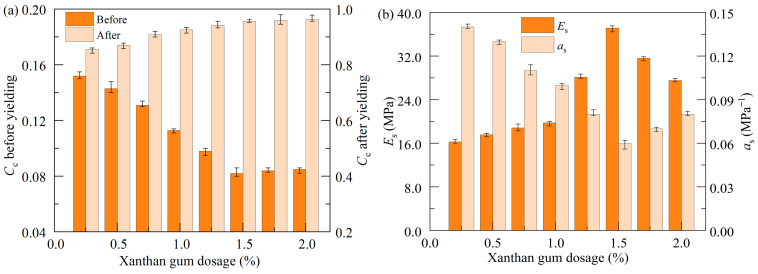
Effect of xanthan gum dosages on soil compressibility: (**a**) compression index; (**b**) modulus and coefficient of compressibility.

The compressibility of soil can be evaluated by various parameters including the compression index, modulus of compressibility (*E_s_*), and coefficient of compressibility (as). Figure 10 illustrates the variations in these parameters of the stabilized soil with different xanthan gum dosages at a 28 d curing age. The pre-yield and post-yield compression indexes are defined as the slopes of the fitting lines representing the two segments of the consolidation curves before and after the yielding points in Figure 9a. The modulus of compressibility and coefficient of compressibility are derived from the changes in void ratio as the vertical pressure increases from 100 kPa to 200 kPa in a pressure range before yielding. One can note that, when the vertical pressure is less than the yield stress, the compression index (*C_r_*) and coefficient of compressibility are small while the modulus of compressibility is large, showing desirable effectiveness of xanthan gum in reducing the compressibility of soft soil. Once the vertical pressure reaches the yield stress, there is a drastic increase in soil compressibility, which is characterized by a significant increase in compression index (*C_c_*). This phenomenon is associated with a sudden breakup of the cementation bonds and the brittle failure of the stabilized specimen, as illustrated in Figure 5a and Figure 6b. Venda Oliveira et al. [40] observed similar findings in their study on the compressive behavior of cement-stabilized soft soil.

It is noteworthy that, before yielding, with an increase in xanthan gum dosage, the compression index and coefficient of compressibility exhibit an initial decrease followed by a slight increase, with a turning point at a xanthan gum dosage of 1.5%. Meanwhile, the modulus of compressibility initially increases and then decreases, showing a reverse trend. At the optimal dosage (1.5%), the modulus of compressibility is enhanced to 37.13 MPa before yielding, whereas the corresponding compression index and coefficient of compressibility are reduced to 0.082 and 0.061 MPa^−1^, respectively. These data suggest that xanthan gum has modified soft soil to be a foundation material with low compressibility, referring to the Chinese technical code GB50007-2011 [41]. The above findings are consistent with the previous observations that an appropriate addition of xanthan gum notably reduces soil ductility, making the soil more brittle (Figure 6b).

### 3.3. Physicochemical Properties

#### 3.3.1. pH Value

**Figure 11:**
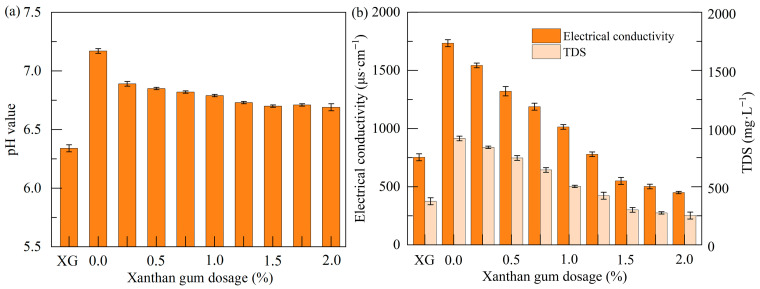
Effect of xanthan gum dosages on physicochemical properties: (**a**) pH value; (**b**) electrical conductivity and TDS.

Figure 11a shows the pH values of xanthan gum, untreated soft soil, and stabilized soil. The term “XG” in this figure represents xanthan gum. It is observed that the leachate of xanthan gum displays weak acidity, whereas the leachate of the untreated soft soil is weakly alkaline. Compared to the untreated soft soil, the stabilized soil exhibits some changes in pH, although these changes are not significant. Specifically, with an increase in xanthan gum dosage, the stabilized soil shows a trend toward weak acidity, with pH values ranging between 6.6 and 6.9. This acidic condition could provide a favorable environment for the reaction between xanthan gum and soft soil, contributing to soil stabilization.

#### 3.3.2. Electrical Conductivity and TDS

The electrical conductivity and TDS of xanthan gum, soft soil, and the stabilized soil are illustrated in Figure 11b. These two parameters typically reflect the amount of free ions and dissolved solids in the soil leachate. On one hand, the electrical conductivity of the soil leachate shows a significant decrease with an increase in xanthan gum dosage. This phenomenon implies that xanthan gum interacts with certain free ions in the soil leachate, which reduces the electrical conductivity. On the other hand, the TDS results reveal that an increase in xanthan gum dosage leads to a gradual decrease in the amount of dissolved solids in the soil leachate. These findings suggest that the structure of the soft soil after stabilization becomes more stable, exhibiting stronger water stability.

### 3.4. Functional Groups and Microstructure

#### 3.4.1. FTIR Analysis

**Figure 12:**
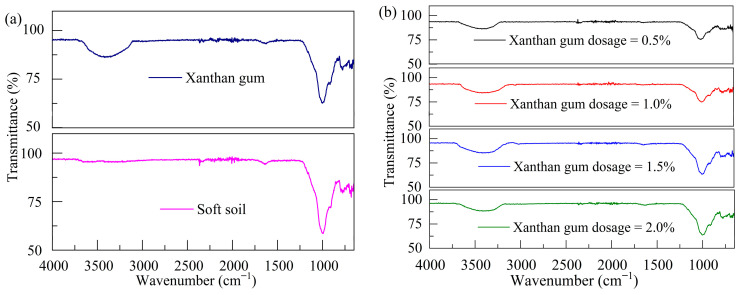
FTIR spectra of xanthan gum, soft soil, and stabilized soil: (**a**) soft soil and xanthan gum; (**b**) stabilized soil.

Figure 12 illustrates the results of the FTIR tests. It is observed that xanthan gum displays a strong characteristic absorption band at 3421 cm^−1^ attributed to the stretching vibration of the hydrogen–oxygen bond (O-H). Moreover, a characteristic absorption peak emerges at 1668 cm^−1^, arising from the stretching vibration of the carbon–oxygen double bond (C=O). Furthermore, in the fingerprint region (1300–600 cm^−1^), two distinct absorption peaks at 1006 cm^−1^ and 781 cm^−1^ are observed, representing the bending vibration of the carbon–oxygen bond (C-O). These peaks are closely associated with the characteristics of xanthan gum as an organic polymer carbohydrate. The above results suggest that the molecular chain of xanthan gum is abundant in hydroxyl groups (-OH) and carboxyl groups (-COOH). These functional groups facilitate the hydrogen bonding reactions between xanthan gum and water molecules and contribute to the formation of a colloidal solution in water, ultimately leading to the creation of a high-viscosity xanthan gum gel. Absorption peaks of some chemical bonds such as C=O (1653 cm^−1^), Si-O-Si (997 cm^−1^), and C-O (775 cm^−1^) are observed in soft soil. This indicates that soft clay contains organic functional groups that are highly reactive and capable of undergoing chemical reactions with the functional groups in xanthan gum. These reactions not only modify the structure of soft soil but also significantly improve its mechanical properties.

A comparison of the FTIR results before and after soil stabilization reveals no obvious changes in the FTIR spectra due to the significant overlap in the functional groups shared by both soft soil and xanthan gum. Despite this overall similarity, a notable difference emerges: the occurrence of a broad peak at 3382 cm^−1^ in the stabilized soft soil. This peak arises from the shift of the characteristic peak originally observed at 3421 cm^−1^ in xanthan gum, resulting from hydrogen bonding and the stretching vibration of -OH groups.

#### 3.4.2. SEM-EDS Analysis

**Figure 13:**
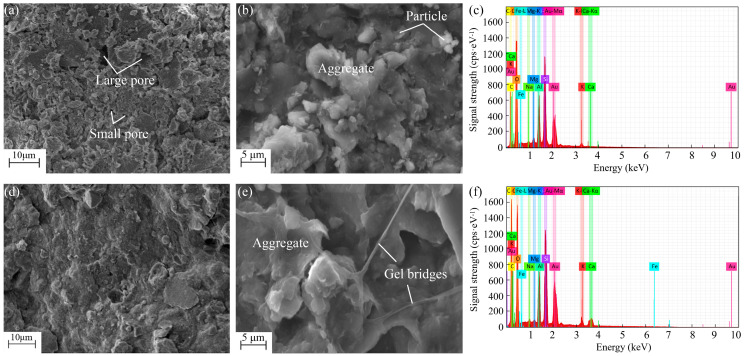
SEM micrographs and EDS patterns of the soil: (**a**–**c**) untreated soil; (**d**–**f**) stabilized soil.

Figure 13 presents the SEM micrographs and EDS patterns of the untreated and stabilized soil. The untreated soil primarily consists of numerous dispersed soil particles, along with a few aggregates resulting from the agglomeration of soil particles (Figure 13a,b). These dispersed soil particles appear sheet-like and mainly exhibit point-to-point and point-to-face contacts. Figure 13a also reveals the presence of many large pores between soil aggregates and numerous small pores between dispersed soil particles or soil particles and aggregates, suggesting a loose microstructure of the untreated soil. Consequently, the untreated soil exhibits low strength while high compressibility at the macroscopic level.

Compared with untreated soft soil, the stabilized soil exhibits distinct microscopic morphology (Figure 13d,e). Firstly, xanthan gum gel wraps soil particles and serves as “glue” or “bridges” to bond separated soil particles together because of its high viscosity, resulting in the formation of numerous large aggregates. Secondly, xanthan gum gel fills the previously existing large pores between soil aggregates, thereby reducing soil void ratio and enhancing the integrity and stability of the soil structure. These microscopic characteristics necessarily improve the strength and durability of the soil while reducing its compressibility (Figure 6, Figure 7 and Figure 10).

Figure 13c shows that the surface of the untreated soft soil exhibits numerous positively charged metal ions (e.g., Ca^2+^, Na^+^, Al^3+^, K^+^, and Mg^2+^). This is due to the dissociation of the hydroxyl groups (-OH) and carboxyl groups (-COOH) on the surface of soil particles, which become negatively charged and can attract metal ions from the surrounding environment. Upon stabilization with xanthan gum, there is no significant change in the elemental composition, except for minor variations in the proportion of specific elements, such as a slight increase in the proportions of C and O elements (Figure 13f). This phenomenon is attributed to the formation of hydrogen bonds between water molecules and the -OH and -COOH groups present on the molecular chain of xanthan gum. These hydrogen bonds facilitate interactions between xanthan gum and soil particles, thereby significantly enhancing the water stability of the soil. This enhancement is characterized by notable reductions in electrical conductivity and TDS in the stabilized soil, as illustrated in Figure 11b.

## 4. Discussion

**Figure 14:**
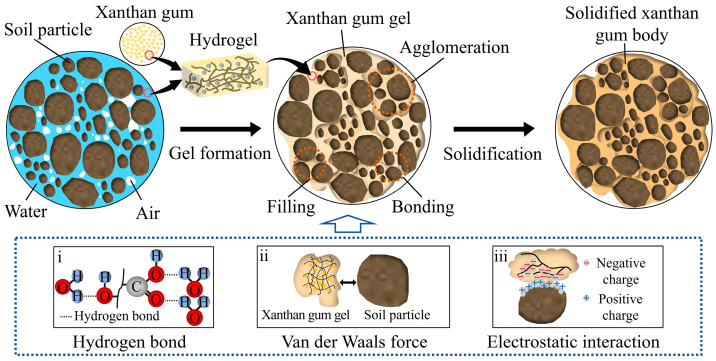
Mechanism behind the stabilization of soft soil using xanthan gum: (**i**) hydrogen bond; (**ii**) van der waals force; (**iii**) Electrostatic interaction.

Based on the experimental results above and the characteristics of both xanthan gum and soft soil, the mechanism behind the stabilization of soft soil with xanthan gum can be explored (Figure 14), with primary focus on the following three aspects:

(1) *Mechanism behind the bonding between xanthan gum and soil particles*. When xanthan gum is added to soft soil, the hydroxyl (-OH) and carboxyl (-COOH) functional groups in the molecular chains of xanthan gum interact with water molecules in soft soil through hydrogen bonding, forming a hydrated layer around xanthan gum molecules. This process allows the xanthan gum molecules to expand in water, further increasing their viscosity, and facilitate the production of hydrogels (Figure 14). Meanwhile, strong hydrogen bonding forces and van der Waals forces exist between the molecular chains of xanthan gum [25,35]. Under the combined action of these forces, the xanthan gum gel (hydrogel) formed in water possesses a stable three-dimensional network structure. This structure efficiently absorbs and retains water while limiting the movement of molecular chains of xanthan gum gel, thereby enhancing its viscosity. Moreover, charged groups carried by the molecular chains of xanthan gum gel interact with oppositely charged groups on soil particles under electrostatic forces [19,23]. This interaction facilitates the adsorption of xanthan gum molecules on soil particles, enhancing the bonding strength between the xanthan gum gel and soil particles. Additionally, van der Waals forces exist between xanthan gum molecules and fine soil particles [42], which also contribute to the connection between them. Therefore, xanthan gum gel can firmly adsorb on soil particles and bring soil particles together due to the abovementioned microscopic forces. Since soft soil is weakly alkaline and xanthan gum is weakly acidic, a neutralization reaction occurs when they are mixed together, resulting in a decrease in alkalinity of soft soil, or even turning it slightly acidic, as shown in Figure 11a.
(2) *Mechanism behind the reinforcing effect of xanthan gum on soil*. Xanthan gum gel possesses high viscosity and mechanical strength due to its unique molecular structure and intermolecular interactions, including van der Waals forces, hydrogen bonds, and electrostatic interactions [22]. When xanthan gum is mixed into soft soil, the resulting xanthan gum gel effectively fills the spaces between soil particles, thereby reducing the void ratio of the soil (Figure 9a). The filling of xanthan gum gel also prevents the migration and accumulation of water in soil pores. These enhancements not only significantly increase the compressive strength of the soil but also reduce its compressibility [19]. Moreover, the molecular chains of xanthan gum gel can act as “glue” or “bridges” under van der Waals forces and electrostatic interaction, tightly bonding loose soil particles together to form large aggregates (Figure 13). As a result, the structural integrity of the soil is greatly enhanced, further improving its strength and stability while reducing its ductility. The influence of xanthan gum on soil mechanical behavior is closely related to the dosage. When the xanthan gum dosage is too low, the filling and bonding effects provided by xanthan gum gel are weak, thus failing to effectively improve the behavior of soft soil. However, when the dosage is excessive, the xanthan gum gel wrapping around soil particles becomes too thick, reducing the effective contact between soil particles, which is detrimental to the performance of soft soil. Therefore, only with an appropriate dosage of xanthan gum can its role be fully exerted, maximizing the improvement in soil mechanical properties (Figure 6, Figure 7 and Figure 10).
(3) *Mechanism behind the solidification of xanthan-gum-stabilized soil.* The xanthan-gum-stabilized soft soil gradually solidifies during the curing process. Firstly, as the curing age increases, the hydration reaction of xanthan gum becomes increasingly complete, leading to the formation of more cross-linking points between its molecular chains. This results in the development of a more stable three-dimensional network structure. Consequently, the viscosity of the xanthan gum gel increases, enabling it to more effectively bond soil particles together. Secondly, xanthan gum continuously absorbs water from soft soil, reducing its water content by forming a hydration layer. At the same time, the filling effect of the xanthan gum gel within the soil pores becomes more pronounced [20], progressively increasing the soil density. Furthermore, the entanglement and intertwining between the molecular chains of xanthan gum gel and soil particles restrict the movement of soil particles, considerably improving the integrity of soft soil. These effects collectively promote the solidification of the stabilized soil, continuously enhancing the strength and stability of the soil while reducing its compressibility. For this reason, the UCS of the stabilized soil shows a positive correlation with the curing age, as shown in Figure 6. Additionally, because an appropriate amount of xanthan gum is effective in reducing soil void ratios and enhancing soil structural stability, it can effectively resist structural damage of the soil. Consequently, xanthan gum notably delays the strength loss of the stabilized soil under dry–wet cycles, exhibiting significantly better durability than the untreated soft soil.

Compared to general clayey soils, soft soil exhibits poor mechanical behavior primarily due to its extremely high water content and large void ratio. Adding xanthan gum can effectively enhance the strength and deformation resistance of soft soil by addressing these issues through complex physicochemical reactions. Firstly, xanthan gum significantly absorbs excess water from soft soil and converts free water into stabilized water, thereby obviously reducing its water content. At the same time, xanthan gum forms a gel with a stable structure in soft soil, effectively filling the pore space and reducing the void ratio of the soil. This ultimately improves the soil strength and modulus. Furthermore, the molecular chains of xanthan gum are able to tightly intertwine with fine soil particles and strongly interact with them, which enhances the integrity of the soil. Finally, as a natural high-molecular-weight biopolymer, xanthan gum is environmentally friendly and can continuously improve the strength and deformation resistance of soft soil without causing negative impacts on the ecological environment. Therefore, xanthan gum shows great promise as a biopolymer for the stabilization of soft soil.

## 5. Conclusions

The UCS, durability, and compressibility of xanthan-gum-stabilized soft soil were analyzed through mechanical tests, with the stabilization mechanism revealed by physicochemical analysis and microscopic observations. This study confirmed the effectiveness of using xanthan gum for soft soil stabilization. The following conclusions can be drawn:

(1) The UCS of the stabilized soil gradually increases with curing age, highlighting a notable advantage of xanthan gum in promoting long-term strength development. With increasing xanthan gum dosage, the UCS exhibits an initial significant increase followed by a slight decrease. The latter decrease is likely attributed to the uneven distribution of xanthan gum at higher dosages. The maximum 28 d UCS (i.e., 376.3 kPa) occurs at a dosage of 1.5%, satisfying the strength requirement for highway foundations.
(2) The UCS of the stabilized soil considerably decreases during the initial four dry–wet cycles and then tends to be stable. The optimal xanthan gum dosage for resisting strength degradation is approximately 1.5%, where the UCS of the stabilized soil drops by only 24.1% after ten dry–wet cycles. Thus, xanthan gum not only increases soil strength but also enhances durability under varying environmental and climatic conditions.
(3) Xanthan gum effectively reduces the void ratio of soft soil, with the consolidation yield stress varying similarly to UCS as the dosage increases. Before yielding, it lowers the compression index and coefficient of compressibility while increasing the modulus of compressibility. At a dosage of 1.5%, the modulus of compressibility is enhanced to 37.13 MPa, demonstrating desirable effectiveness of xanthan gum in reducing the compressibility of soft soil.
(4) The stabilization mechanism of xanthan-gum-treated soft soil primarily lies in the bonding and filling effects of the hydrogel resulting from the hydration of xanthan gum. These effects originate from the hydrogen bonds, van der Waals forces, and electrostatic interactions between xanthan gum molecular chains and soil particles.

These findings indicate that xanthan gum is a promising stabilizing agent for soft soil. The optimal dosage is 1.5% (corresponding to 25.95 kg xanthan gum for 1 m^3^ soft soil), at which the stabilized soil exhibits the highest UCS, best durability, and lowest compressibility. However, this study has certain limitations as it did not include a detailed comparison of performance, cost, and application conditions with conventional stabilizers and only assessed the short-term durability of the stabilized soil. These issues should be addressed in future research.

## Figures and Tables

**Table 1:** Key physical properties of soft soil.

Natural Water Content (%)	Natural Density (g/cm^3^)	pH	Specific Gravity	Plastic Limit (%)	Liquid Limit (%)	Plasticity Index	Organic Matter Content (%)
50.1	1.73	7.17	2.75	22.9	38.5	15.6	1.08

**Table 2:** Fundamental properties of xanthan gum.

Color	State	pH	Viscosity (mPa·s)	Particle Size (Mesh)	Ash Content (%)	Absolute Molecular Mass (g)
Light yellow	Powder	6.34	1593	80	10.1	6.64 × 10^−18^

## Data Availability

The original contributions presented in this study are included in the article. Further inquiries can be directed to the corresponding author.
